# Why do we need agent-based macroeconomics?

**DOI:** 10.1007/s43253-022-00071-w

**Published:** 2022-03-29

**Authors:** Silvano Cincotti, Marco Raberto, Andrea Teglio

**Affiliations:** 1 DIME, University of Genova, Via Opera Pia 15, Genova, 16145 Italy; 2 Department of Economics, Ca’ Foscari University of Venice, Cannaregio 873, Venezia, 30121 State Italy

**Keywords:** Complexity, Agent-based models, Economics, Economic policy, A12, B22, B59, D50, E00, E70

## Abstract

We are entering the third decade of the twenty-first century with profound uncertainties and crucial challenges for the world economy. Phenomena like climate change, digital transformation, migration, demographic changes, and the ongoing COVID pandemic need to be understood and promptly addressed. We argue that the agent-based approach in economics is well suited to tackle these topics, because of its capacity to integrate the “micro” and “macro” dimensions by modelling the network of interactions among heterogeneous economic agents and their aggregate outcomes. This paper explains why the agent-based methodology is needed to overcome the limitations of the neoclassical approach in economics, which has not been able to properly address those challenges. To do so, the paper retraces the main stages of the scientific evolution in a general historical and epistemological perspective, showing how the paradigm of reductionism, which led to extraordinary advances after the scientific revolution of the seventeenth century, is less effective when addressing the main challenges ahead. On the other hand, the sciences of chaos theory and complex systems can provide the economic discipline with more suitable instruments to face those challenges. Finally, the paper briefly presents the contributions of the special issue, which use applications of agent-based models to study the main problems of our times.

## Introduction

We are witnessing uncertain and rapidly changing economic landscapes where some ongoing processes are expected to progress in the years to come, constituting crucial challenges both for the economy and economics as a discipline. First, the climate change issue will need a relevant mobilization of resources to foster the ecological transition, with far-reaching consequences on our business and financial landscapes, still mostly addicted to fossil fuels. Second, the digital transformation could result in tremendous productivity gains but also in widespread unemployment crises. Third, the recent COVID emergency has emphasized the importance to carefully consider the impact of human activity on our ecosystems, which goes well beyond carbon emissions and could increase the likelihood of virus spillovers. Furthermore, the necessity to maintain economic activity alive during lockdowns has surely accelerated the digitalization of most services and routine-based jobs. Finally, there are still relevant questions on a sustainable and inclusive growth able to face demographic changes and migration patterns with the aim to enhance the awareness on opportunities, risks and impacts of policy measures and regulations.

We argue that the agent-based approach in economics is well equipped to tackle these topics, because of its capacity to integrate the “micro” and “macro” aspects by modelling the network of interactions among the economic agents (thus also offering access to the “meso” dimension^1^), and their aggregate outcomes. The objectives of the special issue are to collect and present state-of-the-art contributions in the field, which have been able to provide both novel theoretical foundations and relevant policy advices to cope with the big issues outlined above. A modeling approach that is able to encompass (i) endogenous shocks, (ii) agents’ heterogeneity, (iii) non-market interactions, and (iv) out-of-equilibrium dynamics, is best suited to study the complex evolutionary processes on which global crises are unfolding. These processes are characterized by feedback loops, such as the one between the economy and the environment, tipping points, such as the ones in climate change dynamics, the distributive impact of different climate and welfare policies, and the emergence of phenomena at the aggregate level, which are not obvious from micro behavior.

The paper is organized as follows. In Section 2, we will provide an excursus about the roots of the reductionist approach of modern science in the seventeenth century, its relevant successes but also its more recent crisis due to the very late developments which led to a re-evaluation of the holistic paradigm and to the birth of the so called complexity science. Section 3 presents the agent-based modelling approach as the most appropriate methodology to model complexity and interactions in economics, whereas Section 4 discusses its recent application to macroeconomics and why it provides a superior modelling paradigm with respect to the neoclassical one in addressing both empirical evidence and theoretical issues. Section 5 presents the contributions of this special issue, i.e. some recent and novel applications of agent-based macroeconomics to modelling global crises, such as climate change, financial instability and the pandemic. Finally, conclusions are drawn in Section 6.

## From reductionism to the science of complexity

History of science traces the birth of modern science back to the process of refounding scientific knowledge and the consequent reformulation of the relationships between scientific and philosophical research. The process began in 1200 but took place mainly between the second half of the sixteenth century and the first half of the seventeenth century. In this context, the subject of scientific research is only the physical world and, as in the philosophical tradition, the attention is dedicated to the separation of the aspects of reality from the moral, religious, sentimental and even metaphysical ones.

In modern science, laws are conceived as uniform in space and constant in time, according to an optimistic vision of science itself, which underlies the presumption, questioned only in the mid-eighteenth century by Hume, of an immutable order of nature. With the advent of modern science, research, discovery and elaboration of natural laws are essentially based on experience, hence the empirical and experimental character. Only experience can provide the starting point for formulating theories. The truth of a theory must always be linked to its practical verification, confirming the anti-metaphysical approach of modern science and its refusal to base the knowledge of nature on something that is not attributable to particular experiences.

The laws of modern science are therefore of a mechanical type, they must be expressed in geometric-mathematical terms and fully knowable by human reason; they constitute an objective and neutral knowledge, which is a common heritage and develops thanks to cumulative contributions. These principles were established by Galileo Galilei in the “*Sidereus Nuncius*”, where in 1610 he made public the results of his astronomical research, indicating precisely the used methods and instruments of observation, so that his experiences could be known and replicable. Later, with the “*Dialogo sopra i due massimi sistemi del mondo*” of 1632, he formalized the experimental method based on hypothesis and verification, as a basis for formulating theories, and consolidated the process of mathematizing physics. The attitude of trust in the means with which modern science confronts nature, already present in Leonardo da Vinci’s research, therefore reaches systematicity in Galileo’s thought.

The laws discovered by modern science define exclusively causal relationships between natural phenomena, excluding any metaphysical reference. To know nature and predict its evolution, science exerts a separation of the part from the whole, referring only to the quantitative aspects of material reality (that is those that can be mathematically measured) and considering the qualitative ones as secondary and negligible. In doing so, modern science reduces Aristotle’s four causes to the efficient cause alone, eliminating any finalistic explanation. In the construction of this mathematical and mechanistic interpretation of the world, the knowledge of the mathematical models already present in Greek thought (e.g. Pythagoreanism, Euclid’s geometry) is decisive. However, it is only with Descartes, and his “*Discours de la methode*” of 1637, that the manifesto of modern science is established as the analytical method based on the reduction into simple problems.

From Galileo onward, the dominant paradigm in the natural sciences will be reductionism, which rests on solid pillars: (i) phenomena appear complex because they are not considered correctly; (ii) free from ignorance and prejudices, the man learns to read in the great book of nature which “*è scritto in lingua matematica, e i caratteri son triangoli, cerchi, ed altre figure geometriche*”;^2^ (iii) the complexity of nature is only apparent and, to explain it, a few simple laws (mathematical equations) are sufficient. Therefore, only the elementary part becomes fundamental, while everything that derives from the cooperation of many parts has little scientific dignity. When the part considered elementary is in turn broken down, it loses its dignity in favor of its components. Thus, while in the nineteenth century the simple parts were atoms and chemistry was considered the queen science of the intimate structure of matter, in the twentieth century the discovery of elementary particles led Paul Dirac, the positron theorist, to declare with contempt that “Everything else is chemistry”.

The reductionism program can be summarized according to the two following prescriptions:

- give up studying nature as an organic whole and concentrate on simple and quantifiable phenomena by isolating them from everything else;
- disassemble any complex structure by reducing it into parts sufficiently small to be described and understood through simple mathematical laws.

Reductionism and the reductionist view of science have relevant practical applications and have contributed in an essential way to the development not only of modern science, but of human civilization. Knowing nature becomes the necessary condition to be able to predict its evolution and modify it for the benefit of humanity. To some extent, with the advent of modern science, the Renaissance concept that places man at the center of reality takes shape, in a project of human domination of the natural phenomena of which Francis Bacon with his Novum Organum is probably the most aware theorist, the prophet of technological civilization. The law of gravitation that regulates the motion of planets, the laws of thermodynamics that allow the construction of the steam engine and the internal combustion engine, the equations of electromagnetism that are the basis of electrical engineering, optics and modern telecommunications are all impressive advances resulting from the reductionist method. With the birth of modern science, the holistic view of phenomena is relegated out of the domain of science.

The holistic thesis that can be summarized in the expression “the whole is more than the sum of the parts of which it is composed” is a general principle that has been differently articulated in the various disciplines. Within the philosophy of science, holism is often identified as an anti-reductionist position, based on the assumption that the properties of a system cannot be explained solely by its individual components. However, the contraposition, the conceptual dichotomy holism/atomism is a pretext and is philosophically groundless. The holistic approach does not preclude the atomistic and reductionist theory, but assumes that the aggregate can get different and superior performances with respect to the mere sum of the parts, leading to the concept of emergent properties to understand phenomena that the atomistic and reductionist theory cannot clarify, thus placing itself in terms of non-reductionism rather than anti-reductionism. Holism and atomism simply deal with “different levels” of aggregation of matter; a matter that at the levels of fermions and bosons has properties that are no longer those of the aggregates formed by these elementary particles (be they first atoms, then molecules, then macromolecules up to living beings).

It is interesting (if not obvious) to observe that the very development of modern science has led to the crisis of reductionism itself and to a re-evaluation of the holistic paradigm. The reductionist vision has in fact begun to show dangerous cracks with the awareness that once the building was dismantled it was not obvious to rebuild it starting from the fundamental bricks. Already in the nineteenth century, statistical mechanics, founded by Ludwig E. Boltzmann, highlighted an irremediable paradox: the irreversibility of macroscopic phenomena is neither deducible from microscopic laws nor compatible with their reversibility (the time arrow problem). At the turn of the 1800s and 1900s, Henri Poincaré shows how even the motion of only three point-like bodies with gravitational interactions is complex and unpredictable despite the simple equations from which it is derived. This opened the door to the theory of deterministic chaos and more generally to the strong dependence on the initial conditions of non-linear systems. In the 1900s, the physics of matter brought to the fore the collective and irreducible character of many natural phenomena: magnetism, super-conductivity, superfluidity, etc. The very concepts of order and disorder were understood to be emergent properties of aggregate systems rather than of the individual parts that made them up. These evidences led to an increasing awareness of the application limits of the reductionist approach, which is adequate only for the study of systems that are reducible on a logical basis to a series of semi-isolated subsystems and are only weakly interacting with the outside (low coupling). Examples in natural systems are the gravitational coupling of the Sun-Earth-Jupiter, the dynamics of rarefied (perfect) gases, etc. For low-coupling systems, the analytical representation of the aggregate requires an infinitely smaller number of variables than is necessary for the description of its components; the reciprocal semi-isolation as well as the weak relationship with the external world make the descriptive equations (algebraic rather than differential), generally linear, few and simple. The linearity of the equations and the low coupling condition make applicable the principle of superposition of the effects, which according to the reductionist vision foresees that the effect of a sum of input perturbations is equivalent to the sum of the effects produced by each single isolated perturbation. Many of the scientific successes of reductionism consist of linear systems that satisfy the superposition principle: electromagnetism (Maxwell and Laplace equations), wave propagation (d’Alembert’s equations), diffusion (heat equations), etc. Even the Schrodinger equation is linear.

Conversely, a highly complex system is not reducible to the sum of its subsystems. It is always possible to identify and represent the subsystems that make up a highly complex system, but their nature as open systems, strongly interacting with each other and with the external environment does not allow a reduced analytical representation of the aggregate through a few simple descriptive relationships. The equations that link their dynamic variables are generally non-linear and their temporal evolution is not always univocally determinable (although formulated by means of deterministic equations). On the one hand, we have strongly non-linear systems, possibly even low-dimensional systems, in which the evolutionary indeterminacy is intrinsic to the non-linearity of the equations that govern them and is strongly influenced by the initial conditions or by even differential perturbations. The theory of chaotic systems was born in the 1970s of the twentieth century (even if, as already mentioned, Henri Poincarè had already advanced a definition of deterministic chaos already at the turn of the 1800s and 1900s) and finds in Mandelbrot’s fractals as well as in the butterfly effect of Lorenz’s attractor its own archetypes for the general public thanks to the popular work *Chaos: Making a New Science* published by James Gleick in 1987.

On the other hand, we have highly complex systems composed of a large number of interacting elements with even simple local interactions. These systems are “adaptive”, that is they are able to receive information and stimuli from the outside world and to adapt their evolution accordingly. The collective behavior of the system is to some extent unpredictable and not attributable to the laws of local interaction, it is characterized by emergent properties linked to feedback loops (positive and / or negative) existing in the interactions between subsystems that make up the aggregate system. Typical highly complex and adaptive systems are living beings in general and human systems in particular. Thomas C. Schelling’s 1969 work *Models of Segregation* is a milestone in the study of highly complex social systems, where the system’s outcome differs from the individual intentions, and lays the foundation for the application of agent-based modeling to social, economic and financial systems.

In a short period, a revaluation movement of the holistic approach has quickly involved all fields of knowledge, from the humanities to the natural sciences. This is the context in which it was born the Science of Complexity, which studies those phenomena that cannot be understood if they are analyzed by separating their individual parts. Complexity has historically been a term applied to natural and social phenomena in a generic way but in recent decades it has taken on a technical-scientific meaning. Complexity has become a new paradigm of science and P.W. Anderson published in 1972 the work *More is different*, the manifesto of the Science of Complexity. In it, Anderson retrieves and re-evaluates all the key elements of the holistic paradigm:

- reducing everything to simple fundamental laws does not imply the ability to start from those laws and reconstruct the universe;
- the behavior of large aggregates of elementary particles cannot be explained by simple extrapolation of the properties of a few particles;
- the whole becomes not only more, but also very different from the sum of its parts.

On the thrust of this scientific movement, the Santa Fe Institute was born in 1984 as the first multidisciplinary center for the study of complex systems, with the aim of sharing different research streams in the Science of Complexity.

There are two elements that determine the birth and development of the new science of complexity: (i) the need and the ambition to study phenomena that once were not even recognized and (ii) the development and scientific use of computers. If the first aspect can perhaps be considered intrinsic in human nature, the second finds its reference point in the work *Every planar map is four colorable* by K. Appel, W. Haken and J. Koch (1977), which for the first time demonstrated a mathematical theorem with the help of a computer. From then on, the computer was no longer a simple means of calculation, but became an instrument of scientific investigation.

On top of the so-called Santa Fe approach, which represents one angle, it is also worth mentioning the existence of other approaches to complexity, such as (i) the Brussels–Austin school, led by Prigogine since the 1970s, whose subjects are self-organization and dissipative structure from physics, chemistry and biology (see, e.g. Prigogine (1977)), which points out a new kind of viable order out of chaos, such as life cycle and economic resilience (see also Chen (2019)); and (ii) the computational complexity approach (Velupillai 2010), which advocates a new formalization of mathematical economics and game theory on the basis of a strictly rigorous algorithmic mathematics, to align theory with algorithmic applicability, for experimental and computational exercises.

It is useful to observe how with the advent and development of the reductionist epic, the scientific instrument became the basis for Galilean empiricism, the basis for observing, formulating and verifying theories. The telescope (which approaches objects that are too far away) and the microscope (which magnifies objects that are too small) were the means of the successes of reductionism, analyzers that separate what the eye unites, to understand objects by studying their details. Similarly, the new science of complexity creates its own instrument, the macroscope, which unlike the telescope and microscope is not a physical object. J. De Rosnay in his 1975 work *Le macroscope* offers us a clear definition:

- the macroscope is a symbolic tool made up of methods and techniques taken from very different disciplines;
- It does not serve to see larger or farther, but to observe what is at once too great, too slow and too complex for our eyes.

With the science of complexity, the tool of scientific analysis is therefore a set of methods and techniques, a symbolic tool, which finds its concretization in data, algorithms and computers and which allows us to know, predict, govern what goes beyond the limits of direct human experience, laying the foundations for a new Galilean empiricism, based on computational experiments and capable of bridging the inapplicability of the traditional experimental method, typical of natural (replicable) phenomena, to social (irreplicable) phenomena.

## Complexity and agent-based modelling in economics

The holistic approach to the study of economics, prime example of a real adaptive complex system, has seen a long-standing tradition, predating Anderson’s 1972 work. Herbert A. Simon (to whom we owe the development of decision-making within economic organizations and the very concept of bounded rationality) in his work *Theories of Decision-Making in Economics and Behavioral Science* published in American Economic Review in 1959 argued that “The very complexity that has made a theory of the decision-making process essential has made its construction exceedingly difficult. Most approaches have been piecemeal—now focused on the criteria of choice, now on conflict of interest, now on the formation of expectations. It seemed almost utopian to suppose that we could put together a model of adaptive man that would compare in completeness with the simple model of classical economic man. The sketchiness and incompleteness of the newer proposals has been urged as a compelling reason for clinging to the older theories, however inadequate they are admitted to be. The modern digital computer has changed the situation radically. It provides us with a tool of research—for formulating and testing theories—whose power is commensurate with the complexity of the phenomena we seek to understand. [...] As economics finds it more and more necessary to understand and explain disequilibrium as well as equilibrium, it will find an increasing use for this new tool and for communication with its sister sciences of psychology and sociology.”

Simon’s vision, intrinsically holistic and linked to the future science of complexity, finds further confirmation in his work *The Architecture of Complexity* published in Proceedings of the American Philosophical Society in 1962 where he stated that “Roughly, by a complex system I mean one made up of a large number of parts that interact in a nonsimple way. In such systems, the whole is more than the sum of the parts, not in an ultimate, metaphysical sense, but in the important pragmatic sense that, given the properties of the parts and the laws of their inter- action, it is not a trivial matter to infer the properties of the whole. In the face of complexity, an in-principle reductionist may be at the same time a pragmatic holist.” The modeling approach proposed by Simon outlines a macroeconomic vision based on the interaction of (heterogeneous) agents whose expectations and decision-making processes are based on empirical and psychological intuitions and represents in fact the first formulation of a research program that we now indicate as agent-based macroeconomics. If Simon is the theorist of the holistic approach to the study of economics, Thomas C. Schelling’s 1969 work *Models of segregation* lays the foundations for the real application of agent-based modeling to social, economic and financial systems, applying what is theorized by Simon and sowing the basis of a new line of economic research that in a few years will begin to trace, in the wake of the science of complexity, a new way to tackle the study of economics with computational models based on interacting agents.

## Agent-based macroeconomics

The second half of the 1980s^3^ saw the birth of the agent-based computational economics which grew rapidly in the 1990s, proving to be a suitable approach for studying economic-financial systems as complex adaptive systems. Agent-based computational economics finds the fertile ground for its progress in the availability of low-cost computing power of those years, which made it possible to model the interactions of a large number of heterogeneous agents with limited rationality and conduct computational research. By seizing opportunities and positive feedbacks from other disciplines (e.g. mathematics, physics, engineering, biology, computer science) agent-based computational economics offers economists the opportunity to find answers to dissatisfaction with approaches based on the hypothesis of rational representative agent and on the theory of equilibrium. In fact, it allows the study of aggregate and emerging statistical regularities in the economy, which cannot originate from the behavior of a “typical”, that is “representative” or “average” individual, (see A. Kirman, “Whom or what does the representative individual represent ?”, Journal of Economic Perspectives, 1992 ), but they are the result of the behavior and interaction of heterogeneous agents. The agent-based methodology directly addresses the interaction and coordination processes of economic agents considering the heterogeneity,^4^ adaptation and aggregation of behaviors as the foundations of aggregate and emerging statistical regularities in the economy. Therefore, agent-based economics represents economic-financial systems as sets of interacting heterogeneous agents (with populations from a few units to billions of agents) characterized by internal states, behavioral rules and decision-making processes, interacting with each other and with the environment (interactions both local and system-wide). The interaction network plays a fundamental role and interactions, according to the science of complexity, produce aggregate phenomena intrinsically different from individual behavior.

Agent-based economics models and constructs artificial economies on computers where one can perform computational experiments (what-if analysis^5^) by adopting Galileo’s experimental scientific method also in the context of social sciences. The discipline initially focused on the microeconomic field, with the modeling and analysis of single isolated markets (for example for the stock market see Palmer et al. 1994; Lux and Marchesi 1999; Raberto et al. 2001, for the fish market see Kirman and Vriend 2000, for the electricity market see Nicolaisen et al.^2001^; Guerci et al. 2005, for price motions see Mandelbrot and Hudson 2004, etc...) then, it underwent a terrific development in the following years, with an enormous variety of models of both real and financial markets that in the mid 2000s led to an impressive evolution: the birth of agent-based macroeconomics.

It should be remembered, however, that since the 1970s, the attention of mainstream macroeconomic research has shifted toward dynamic models of general equilibrium, taken as a reference for macroeconomic studies and economic policy analysis. In their original form, these models are based on the hypotheses of representative agents, rational expectations and equilibrium originating from the optimal inter-temporal behavior of all agents, in full consistency and continuity with the reductionist approach. The well-defined conceptual basis and the relatively parsimonious structure of these models has strongly contributed to the appeal of this approach and has led to an impressive number of works in the scientific literature and the emergence of dynamic stochastic general equilibrium (DSGE) models as a reference for macroeconomic policy.

However, several authors have highlighted problematic aspects associated with the use of DSGE models. For example, Kirman (1992) shows how the aggregate behavior of a heterogeneous set of (optimizing) agents cannot be interpreted as the optimal decision of a representative agent. Furthermore, under reasonable informative assumptions, it is not possible to construct adjustment processes that guarantee convergence towards equilibrium (Kirman 2016). The assumption of stability/self-equilibrating coordination of all agents at the equilibrium, in the line of the Walras/Arrow/Debreu tradition, is therefore a strong assumption even in the case where the equilibrium exists and is unique, a condition not guaranteed in many classes of models. Finally, the hypothesis that all agents have rational expectations about economic dynamics has been criticized as unrealistic and very little experimental and empirical evidence suggests that the evolution of agents’ expectations is consistent with the assumption of rational expectations (see, e.g. Carroll 2003; Hommes et al. 2004).

The approach to agent-based macroeconomic modeling, which began to develop in the early 2000s, differs in several respects from DSGE models. In agent-based macroeconomic models, different types of heterogeneous agents with behavioral and forecasting rules interact through explicitly represented market protocols. Economic systems are seen as complex adaptive systems where agents are autonomous, that is, there is no top-down centralized coordination or control mechanism. The meso- and macroeconomic variables are determined by the effective aggregation of the behavior of the populations of agents. This bottom-up approach explicitly captures the interaction at the micro level of different types of heterogeneous economic agents and macroscopic phenomena are explained in terms of a multitude of elementary microeconomic variables that interact according to rules and protocols. Agent-based macroeconomic models therefore aim to provide intrinsically microfounded representations, based on individual behaviors and interaction, and to validate the models by comparing the properties at the aggregate level with empirical data. The global dynamics of these models is based on computational experiments without ex-ante hypotheses on the coordination of individual behavior. Computational experiments endogenously generate fluctuations and economic cycles without relying on external shocks (see, e.g. Dosi et al. 2006; Raberto et al. 2008; Cincotti et al. 2010). Agent-based macroeconomic models highlighted, even before the 2008-2009 crisis, the fundamental role of contagion mechanisms^6^ (see, e.g. Battiston et al. 2007) and the interaction between the real and financial sectors as a cause of instability and sudden economic slowdowns (see, e.g. Raberto et al. 2012). These emerging properties, combined with the ability of agent-based macroeconomic models to incorporate a wide range of behavioral assumptions and to represent institutional characteristics^7^ that are relevant for the analysis of actual policy proposals, have fostered the interest of policy makers in agent-based macroeconomics (see, e.g. “Reflections on the nature of monetary policy non-standard measures and finance theory”, Speech by Jean-Claude Trichet, President of the ECB, Opening address at the ECB Central Banking Conference Frankfurt, 18 November 2010^8^; “The dappled world”, Speech given by Andrew G Haldane, Chief Economist, Bank of England, at the GLS Shackle Biennial Memorial Lecture, 10 November 2016^9^; “Agent-based models: a new frontier for macroeconomics?” by Daniel Hinge (2018), on centralbanking.com^10^; two OECD reports (Thurner 2011; OECD 2012) have determined the development of research in this area in general and in agent-based economic policy in particular. On the relevance of agent-based macroeconomics in policy making we also recommend the recent contribution by Cincotti (2021).

Agent-based models have of course some limitations with respect to other macro modelling frameworks, as Richiardi (2017) and Dawid et al. (2019) put forward. The main limitation of agent-based modelling as tools for supporting policy decisions lies in their challenging interaction with data. In state of the art agent-based models, real data are typically used for the calibration of parameters and initial conditions and in the validation phase, in order to measure the performance of the model in reproducing well-known ‘`‘stylized facts”. Bringing agent-based models to data is not an easy task, and the main criticisms focus on the lack of rigour in the current validation and calibration practices, most of which remain qualitative (Platt 2020). Actually, agent-based models are not fit for data driven simulations, and the main reason lies in the endogenous nature of agents’ interactions, which makes it difficult to synchronize the artificial economy of the agent-based model with real data. We might argue that the main strengths of agent-based models, which are endogenous interactions among agents, are also their main weakness when trying to bring them to data. Overcoming the limitations of agent-based models in terms of providing quantitative predictions is for sure a key challenge in the field.

## Why do we need agent-based macroeconomics?

In the Popperian positivist view, scientific theories allow formulating and setting up a research through a form of reasoning that, starting from axioms and hypotheses, derives consequences that can be verified empirically and experimentally. Actually, this view is considered epistemologically simplistic according to the to modern measurement theory,^11^ which claims that even for the “exact” natural sciences there is no objectively correct or true way of measuring, but the measuring and testing equipment itself usually is constructed in a biased way to show us things the way we have conceptualized before. However, according to Popper’s critical rationalism (see Popper (1959)), the goal of science is to formulate a hypothesis, even assumed as mere conjecture (and not necessarily as a true principle, a general law in the sense of physics), and to deduce verifiable or falsifiable consequences. The hypotheses are therefore the preliminary conditions that are assumed will be verified by the systems that are studied and from which, through a chain of deductions / demonstrations, the validity of one or more properties derives. Economic models should adopt a hypothetical-deductive method to explain economic phenomena and in the face of empirical data it should be possible to consider the models as inadequate/unfruitful. As the humorist Arthur Bloch pointed in his Hawkins’ Theory of Progress “Progress is not about replacing the wrong theory for the correct one but replacing it with a wrong, but refined theory”. Recognizing the inability of a theory to reproduce empirical-experimental evidence therefore represents the occasion for the beginning of a new line of research; however, numerous examples show how reluctance and inertia have transformed these moments from occasions of confrontation to strenuous entrenchments.

Modern macro-economists have built increasingly abstract and sophisticated mathematical models. As mentioned in the previous section, studies in the DSGE literature are based on local approximations of the dynamics of the model around a steady state. These approximations allow us to introduce the hypotheses of representative agents, rational expectations and equilibrium, but in the linearization process the global and emerging dynamics of the underlying model are lost. It therefore becomes problematic, if not impossible, to correctly capture global phenomena such as regime changes and large economic fluctuations. As economic cycles and fluctuations in DSGE models are driven by exogenous shocks, it is possible to study the propagation of these shocks but not to take into account the mechanisms that generate them, nor to understand how we could reduce the risk of emergence of large fluctuations, such as economic crises. Nonetheless, until the early 2000s, confidence in classical theoretical models was unchallenged. “The central problem of depression-prevention has been solved”, declared Robert Lucas in his presidential address at the meeting of the American Economic Association in 2003 and in 2004 Ben Bernanke, future president of the FED, celebrated the Great Moderation in the results and economic performance of the last two decades as a consequence of the refinement of the neoclassical models used in economic policy.

The economic crisis that began in 2007, however, has shown the need for an explanation that involves notions such as interactions and networks, counterparty trust and default contagion in credit markets, in a process entirely endogenous to the economic system, which has spread from the financially driven real estate market to the real economy through credit and financial markets. In fact, in DSGE models, the interaction network of the system is lost in the aggregation mechanism of the representative agent and the representative agent hypothesis does not allow the introduction of a realistic financial market. It should be noted that the financial market, unlike all the other markets considered in a macroeconomic context, is characterized by buyers and sellers (supply and demand) who belong to the same category of economic agent (household / trader). The hypothesis of representative agent makes it intrinsically impossible to introduce a realistic financial market because the expectation of a gain from the simultaneous buying and selling action on the financial market of the only representative agent requires the need to hypothesize that the investor is affected by bipolar syndrome in complete contradiction with the hypothesis of rational expectations. It is therefore not surprising that in the standard DSGE models the financial markets are reduced only to zero-coupon bonds and the balance between supply and demand to the inverse and deterministic relationship between price and interest rate, effectively eliminating the financial market from these models.

Starting from P. Krugman’s article *How Did Economists Get It So Wrong?* published in 2009 in The New York Times^12^, economists began to recognize that many (too many) of the assumptions and conditions underlying the standard DSGE models had substantially reduced the ability of these models to offer policy makers adequate responses to the empirical evidence of recent economic crisis. The inability and limitation of DSGE models to represent reality found further confirmation in the work *Macroeconomic model comparisons and forecast competitions* published by V. Wieland, M. Wolters on voxeu.org^13^ in 2012 (see also Wieland and Wolters (2011)). The work compares the empirical data of quarterly annualized economic growth, the forecasts of the ECB Survey of Professional Forecasters (SPF) and the forecasts of 5 DSGE models conditional on the mean nowcast from the SPF. The DSGE models and their inability to generate an endogenous crisis allow the prediction of time series always characterized by a return to equilibrium, while the real data show the succession of an increasingly deep economic recession.

The serious consequences of the crisis that began in 2007 caused a crisis of confidence in the science of economics and the inability to lead to conclusions consistent with empirical data has turned into a crisis of economic theory as well, outlined by the special cover of The Economist of July 18th, 2009, entitled *Modern Economic Theory - where it went wrong and how the crisis is changing it*. This has generated a demand for economic models other than DSGE, in which an explanation for the crisis and economic phenomena could be offered on the basis of reasonable assumptions. The need to give more fruitful accounts of empirical evidence paves the way for an agent-based macroeconomics that offers the possibility of an explanation through aspects such as heterogeneity, interactions, bounded rationality, and endogeneity, so to provide effective policy guidelines (see, e.g. Gallegati (2021)). In the following, we will elaborate on three critical issues in economics, namely, evidence of power laws in distributional data, expectation formation and the need of more realistic, appropriate, fruitful microfoundations, in contrast to the pseudo-microfoundation pretenses of the neoclassical approach. All three issues are interrelated and overlapping. In particular, we will discuss the problems faced by the neoclassical paradigm to deal with these issues and on the solution offered instead by the agent-based modeling approach.

### Power laws in economics

The study of probability and statistics was established between 1600 and 1800, linked to the need to offer answers to profoundly different problems, from gambling to statistics for the nation states.

From the beginning, a question acquired particular importance: how much information is needed to understand the aggregate behavior of a large number of random variables? The answer to this scientific problem begins with Laplace completing the work begun by De Moivre in 1733. Laplace’s work^14^ of 1809 constitutes the milestone of what is now commonly called the Central Limit Theorem (actually there are several central limit theorems) and which offers the necessary and sufficient conditions so that the sum of random variables tends to be distributed according to a Gaussian law for the number of addends that goes to infinity.

There are numerous phenomena that follow the Gaussian distribution. The heights of people, the intensity of the radiation of a light source, thermal noise, are just a few examples and the scientific literature reports an almost infinite set of empirical and theoretical cases distributed according to the Gaussian and to distributions related to it. For this reason, the Gaussian distribution plays a primary role both in probability and statistics and is therefore assumed as a reference in the most varied fields.

At the end of the nineteenth century, Vilfredo Pareto, studying the distribution of tax revenues of various Western countries, found that the income curve follows a law different from the Gaussian. The data collected show a polynomial (inverse) distribution as reported in his 1897 book *Cours d’Économie Politique*. Pareto’s intuition arises from the study of income distribution tables in an attempt to find an approximation of the distribution and leads him to believe (erroneously) that the power law applies to the entire distribution of wealth with a single exponent. Only in 1960 Mandelbrot showed that Pareto’s law for the income curve applies only to large estates, but even if the polynomial distribution was limited only to the tail, it should be noted that with Pareto a non-Gaussian asymptotic convergence distribution emerges. In fact, the Gauss distribution, with its exponential decay, tends to zero faster than any inverse power and is therefore unable to represent the empirical evidence found by Pareto. From that moment on, countless studies have demonstrated the presence of power-law and similar heavy-tailed distributions in broader contexts, also facilitated by the relatively simple opportunity to identify them: in a log-log-scale probability distribution diagram, power-law distributions correspond to a linear relationship. It is useful to note how this feature could be at the very basis of the work of Pareto, an engineer using the slide rule who bases its operation on logarithmic conversion.

Power-law distributions have been verified in the size of cities (Zipf’s law), as well as in the words used in the texts, social relations, the orders of nodes in scale-free networks, access to internet sites, etc. Without fear of generalizing, almost everything that sees the human being as central seems to follow power laws and in this sense economics and finance, which are social sciences, also respond to the call. In addition to the income distribution curves studied by Pareto, the size, capitalization and number of employees of firms follow power in the upper tail^15^ as well as the returns of a financial market, etc. (see, e.g. Mantegna and Stanley (1995), Axtell (2001), and Erlingsson et al. (2013)).

Power-law distributions are therefore an empirical datum, and an economic model should be able to present an explanation for this phenomenon. Understanding the conditions for obtaining non-Gaussian distributions requires understanding the necessary and sufficient conditions for which the sum of a large number of random variables tends to be distributed according to a Gaussian law. Only by violating these conditions we will be able to understand what conditions may be necessary to have non-Gaussian distributions.

The previously mentioned works of De Moivre and Laplace formulate the central limit theorem in its simplest (and even less general) version relating to similar random variables. Jarl Waldemar Lindeberg in 1922, with the work “Eine neue Herleitung des Exponentialgesetzes in der Wahrscheinlichkeitsrechnung” published in Mathematische Zeitschrift, and Paul Pierre Lévy, with the work “Propriétés asymptotiques des sommes de variables aléatoires enchantées” published in 1935 in the Bulletin des Sciences Mathématiques, resume and generalize the De Moivre-Laplace central limit theorem leading it to the formulation we know today:

*let X1, X2,.... be independent and identically distributed random variables with finite mean and variance, the random variable obtained from the sum of the variables X1, X2,.... converges to the Gaussian distribution*

In order to have a non-Gaussian asymptotic distribution it is then necessary to violate the hypotheses of the Lindeberg-Lévy central limit theorem, i.e. at least one of the following conditions:

- independent random variables;
- identically distributed random variables;
- random variables with finite mean and variance.

Condition c, which is relevant from a theoretical point of view, has a limited practical value.^16^ Since our context refers to the real economic world, by its nature based on finite (and generally scarce) resources, it is not a priori possible to assume its negation as a necessary condition to explain the empirical evidence of power-law probability distributions in economics and finance.

Conversely, the other two conditions represent relevant elements from an agent-based macroeconomic perspective. Condition a. in fact makes it possible to violate the asymptotic Gaussian distribution in the case of dependent random variables, in other words variables linked to each other by a relationship. An economic model that can reproduce this stylized fact through the condition of dependence of the random variables must therefore necessarily consider the presence of an interaction network between economic agents of the same category. As already pointed out, this condition is incompatible with the hypothesis of representative agent as it eliminates by definition the interaction network of the system. It should be remembered that Lévy himself in his 1935 work provided the necessary and sufficient conditions for the central limit theorem to exist in the case of a specific class of dependent random variables, the martingales. Lévy’s central theorem for dependent random variables thus demonstrates the assumption: violating even one of the conditions of the Lindeberg-Lévy central limit theorem makes it possible to have asymptotic distributions with power laws. For instance, path dependence between agents’ choices, leading everyone to make the same choice in short succession clearly violates condition a. and can absolutely give you heavy tails.

Condition b allows to violate the asymptotic Gaussian distribution in the case of differently distributed random variables. Unlike the dependence condition, this aspect has not theoretical depth today in the scientific literature. Nonetheless, it assumes great importance in the context of agent-based macroeconomics because in order to have differently distributed random variables, it is necessary to have a plurality of heterogeneous agents. The heterogeneity of the agents is therefore also a reasonable condition for having non-Gaussian distributions.

With minimal generalization, it can be intuitively argued that violating both conditions a and b can represent a sufficient condition to reproduce the stylized fact of non-Gaussian distributions. An economic model that can offer the possibility of an explanation of distributions with power laws^17^ should, consequently, represent the economic system as a set of interacting^18^ heterogeneous agents, which is the basic hypothesis of agent-based macroeconomics.

Finally, it is also worth mentioning that in macroeconomics it is not only a matter of power laws, but of fat-tailed distributions associated to both mild recessions and deep crises (see, e.g. Stiglitz (2011, 2015)), see also the empirical evidence provided by Fagiolo et al. (2008). In fact, DSGE models are not able to account for this stylized fact (Ascari et al. 2015) which is naturally reproduced by macro ABMs.

### Expectations and formation of economic expectations

The expectation of an economic variable is the value that the variable is expected to assume at a time (or period) subsequent to the one in which the forecast is made. An expectation is said to be fulfilled when the actual variable takes on the expected value. In order for the expectations of all operators to be realized, it is necessary that all formulate the same forecast. According to J.M. Keynes, the formulation of economic expectations resembles those prize games in which participants must choose the 6 most interesting faces among hundreds of photographs and the prize is awarded to those who make the choice closest to the average preferences of the other competitors, who of course face the same question. In formulating an expectation, then, the problem is not guessing what the average opinion is, but what the average opinion expects the average opinion to be. This being the case, according to Keynes, any formalized theory of expectations risks being largely disproved by the facts.

Cagan (1956) and Friedman (1957) focused on empirical ways of changing expectations, introducing the hypothesis of adaptive expectations. Assuming that the variable to be predicted is the inflation rate, the hypothesis of adaptive expectations implies a revision of inflation expectations based on the error made in the present period, i.e. on the difference between actual inflation and expected inflation at present. When the economy is hit by a shock and therefore finds itself outside the so-called steady state, the adaptive modality of revision of expectations always implies systematic errors, because the operators look only at the past values of the variable on which they must form the expectation, without exploiting other information that they also have. This comes at a cost: e.g. firms that form wrong inflation expectations may decide to employ more (or fewer) workers than they should employ to maximize profits; trade unions could negotiate a nominal wage lower than what would allow them to optimize the satisfaction of their members, etc.

To avoid the defects of the adaptive expectations hypothesis, the radically different one of rational expectations was formulated (Muth, 1961), subsequently adopted both in the new classical macroeconomics of the 1970s and 1980s (see, e.g. Lucas), and in the new Keynesian macroeconomics. According to this hypothesis, the economic operators make the best use of all the information available at the moment in which they have to formulate their forecasts. This also includes the information contained in the equations that make up the economic model used to explain and describe the phenomenon on which they form their expectations. If the relevant model of the economy is not deterministic but stochastic (i.e. the economy is marked by random and unpredictable shocks), rational expectations are not translated into perfect forecasts, but in averaged correct forecasts. In any case, forecasting errors would not be systematic, that is no temporal correlation could be identified among the errors. If there was a correlation between errors, moreover, this would be part of the information available to operators and they could exploit it to form predictions.

For example, the rational expectation about the value that inflation will assume is the expected value of the inflation rate, conditional on the information available at present. This means assuming that an individual’s inflation expectation, based on the probabilities that individual subjectively attributes to the various possible realizations of the inflation rate, coincides with the expected value of the inflation rate itself, or with the average of its distribution of objective probability.

According to the hypothesis of rational expectations, each economic operator behaves as if she/he knew the true model of the economy, as if everyone shared it and were aware of the fact that others share it and were able to solve it in order to obtain useful information for forecasting purposes. Only in this way it is possible to circumvent the problem of the beauty contest, highlighted by Keynes, and to ensure that expectations are homogeneous. If, on the other hand, operators (just like economists) believe in different theoretical models, the forecasting strategies will also be different and heterogeneous expectations will emerge.

The hypothesis of rational expectations, as a description of the way in which operators form their expectations, appeared less plausible in 2011 than it was 40 years earlier. It is shaken by the frequent crises of financial markets, which seem to disprove the hypothesis of efficient markets, in turn closely linked to that of rational expectations, in conjunction with the assumption that all operators have the appropriate incentives to seek information able to formulate correct forecasts on the prices of financial assets. A realistic alternative to rational expectations is the one of local information diffusion and herd behavior models (see e.g. Ponta et al. (2011) and Ponta and Cincotti (2018)).

Finally, it is worth noting that heuristics can work better than more sophisticated expectations in truly uncertain worlds, in line with the seminal contributions of Herbert Simon. The key reference here is Gigerenzer and Brighton (2009). Dosi et al. (2020) show in a macro ABM that heuristics outperform OLS forecasts and stabilize macroeconomic dynamics.

### Microfoundation in macroeconomics

The profound disenchantment with neoclassical economic theory and the never resolved problem of the relationship between micro- and macro-economics were central themes of the debate in the early 1990s with a focus on the rejection by macroeconomists of the theoretical results that showed how in context of standard hypotheses on single economic agents it was not possible to reach any conclusion in relation to the uniqueness or stability of the equilibrium (see Sonnenschein (1972), Mantel (1974), and Debreu (1974)). However, it should be noted that, precisely in accordance with the reductionist theory from which the neoclassical models derive, these two characteristics play a central role in modern economic analysis. The possibility of having heterogeneous agents therefore remains a purely abstract concept and the theoretical way out of this dilemma consists in arguing that the behavior of the economy as a whole can be assimilated to that of an individual (“a representative agent”) and at the same time assume that the economy is in equilibrium. Furthermore, to overcome the problem of aggregating heterogeneous agents it is necessary to assume homogeneity of the agents, bringing the representative agent back to a mere aggregation of agents all equal to each other and equal to the aggregate. These assumptions make it possible to eliminate the theoretical difficulty of effectively aggregating heterogeneous agents and also to show how an economy would organize itself in an equilibrium. Unfortunately, the “Weak Axiom of Revealed Preferences”, essential element of the individual choice of Arrow’s “Impossibility Theorem” shows how individual preferences cannot be aggregated into social preferences that satisfy the assumptions made about individuals, explicitly highlighting how the assumption that the properties of an aggregate function reflect those of the individual function has no theoretical basis. See Gallegati and Kirman (2019) for more details.

In the Arrow-Debreu model, agents interact only through the price system and this removes much of the interest of the model as a description of a real economy. Game theory, on the other hand, has tended to limit itself to very limited cases where agents interact directly but strategically in a very sophisticated way. So, one way to see the path taken by many of the scholars of the agent-based research community is to search for an intermediate route. One where agents interact directly, but possibly locally with each other, and where they reason on a much less elaborate level than individuals in theoretical game models are assumed to do. In macroeconomic models based on agents, different types of heterogeneous agents endowed with behavioral and forecasting rules that interact through explicitly represented market protocols and meso- and macroeconomic variables are determined by the effective aggregation of the behavior of agent populations (see, e.g. Battiston et al. (2012)).

Among the most relevant agent-based models of the macroeconomy developed so far, it is worth mentioning the well-known Eurace model^19^ (see, e.g. Mazzocchetti et al. (2018), Ponta et al. (2018), and Bertani et al. (2021)) as well as the branch developed at the University of Bielefeld (Dawid et al. 2018); the Schumpeter meeting Keynes model (Dosi et al. 2010) and its further enrichments to address several policy issues, e.g. the ecological transition (Lamperti et al. 2018) and labor market reforms (Dosi et al. 2020), and finally the series of models developed by the Ancona/Milano groups of scholars which have been pioneers in the field (see e.g. Delli Gatti et al. (2008, 2011); Delli Gatti 2018). General introduction and comprehensive reviews of agent-based macromodels are available in (Fagiolo and Roventini 2017; Dawid and Delli Gatti 2018; Caverzasi and Russo 2018; Haldane and Turrell 2019; Dosi and Roventini 2019). It is also worth mentioning two recent special issues of leading scholarly journals dedicated to the field, i.e. Industrial and Corporate Change, vol. 27 (2018), and Journal of Evolutionary Economics, vol. 29 (2019).

## The contributions of the special issue

All the papers included in this special issue deal with several of the main problems characterizing our era of global crises. Dawid et al. and Nieddu et al. analyze the consequences of innovation (or digitalization) on unemployment and inequality. Guerini et al., Gaffeo et al., and Vigliarolo address topics concerning financialization, the financial crisis, and the effectiveness of monetary policies under different perspectives. Monasterolo et al. and Nieddu et al. tackle climate change related topics, while Vidal-Tomas et al. study the effects of COVID-19 and panic spreading on financial markets.

Methodologies and scopes may differ across papers but they all share the idea that interaction among agents and distributional analysis are needed to better understand the underlying phenomena. Some papers (Dawid et al., Nieddu et al, Guerini et al.) use well-established macroeconomic agent-based models to perform what-if analysis to explore relevant scenarios related to their research questions. Monasterolo et al. employ an hybrid solution that combines a Stock-Flow Consistent (SFC) behavioural model with heterogeneous agents and sectors of the real economy. Gaffeo et al. exploit the potential of agent-based network models to identify, under an historical perspective, what circumstances may enable the endogenous formation of a clearinghouse. Vigliarolo adopts a conceptual approach to describe the complex socioeconomic interactions behind the financialization of the world economy. Finally, Vidal-Tomas et al. propose an empirical analysis to examine the emergence of financial systemic risk from COVID-19 contagions.

This special issue, therefore, reflects the heterogeneity in terms of analyzed topics and used methodologies that characterize the current state of the art in agent-based focused research. In the following, we provide a short description of each work included in this publication.

In a modelling framework with heterogeneous workers and firms, as well as endogenous technology choices of firms and job learning of workers, Dawid et al. examine and compare the effects of more frequent vs. more substantial productivity increasing innovations. They show that under both scenarios the regime change leads to an increase in heterogeneity of productivity and to increased market concentration. However, these effects emerge according to a different time structure in the two scenarios.

Nieddu et al. present two “case studies” simulated with the agent-based Eurace model. The first concerns the digital transformation of the economy, where the authors study the effects on technological unemployment and market concentration. The second regards the sustainability transition, analyzing the impact of carbon tax and feed-in tariff policies on greenhouse gases emissions. The authors also discuss the generative power of the model and its flexibility in tackling different economic problems.

The effectiveness of climate finance policies is examined by Monasterolo et al. by performing simulations with a SFC behavioural model with heterogeneous agents. In particular, they compare green bonds issuing and carbon tax as measures to finance government’s green subsidies, finding that both policies can give rise to trade-offs in terms of decarbonization of the economy, distributive effects and public debt sustainability.

Guerini et al. study the impact of the adoption of Mark-to-Market accounting standard on financial stability, economic growth and sustainability of public budget. The authors make computational experiments, by running simulations with the “Keynes meeting Shumpeter” macroeconomic agent based model (Dosi et al. 2010), finding that Mark-to-Market standards might generate instability in credit supply because of its procyclical nature. They also examine the potential use of Quantitative Easing to neutralize this procyclicality.

The paper presented by Gaffeo et al. examines the preconditions favouring the endogenous emergence of a clearinghouse from a free banking system. They do it by designing an agent-based model where decentralized agents must decide on the type of payment settlement that they can perform by means of coordinated optimizing behaviours. This contribution reveals how the agent-based methodology can also address questions on economic history by designing different scenarios for counterfactual experiments.

Differently from many other papers in this issue, the work of Vigliarolo is not based on simulations of agent-based models but presents a conceptual analysis on the causes and effects of the economic financialization. The author points out the need to reconvert the utilitarian individual reason into a collective ontological reason as the basis of the economy and proposes a process of economic socialization. To this end, he suggests the definition of an intermediate space between the micro and the macro, defined meso-economics where to introduce cultural “social values” that guide the economic process.

The work of Vidal-Tomas et al. is rooted in the general problem of systemic interconnectivity and on the measures to contain systemic risk. They present an empirical analysis about the correlation between the COVID-19 spread and the financial markets dynamics, and about the response of financial markets to the measures implemented to curb the contagion. They prove that the onset of the virus has caused a sudden and simultaneous fall in financial markets and that the adopted policy measures were not able to calm down investors’ panic.

## Conclusion

This contribution presents some reasons why agent-based economics should be considered as a convenient and promising tool for economic modelling and policy analysis. The mainstream approach in economics imitates the typical methodology used in natural sciences (especially physics) for studying and modelling observed phenomena, i.e. uncovering the underlying laws of nature—often written in the language of mathematics. We argue that the tout-court application of the reductionist approach to social sciences in general, and economics in particular, is seriously questionable. Economies are complex adaptive systems that do not share the same characteristics of those natural phenomena that mechanical physics studied so successfully. Therefore, the science of complexity, which also has been conceived in the field of physics, appears as a much more suitable approach for studying social sciences, and economics among them.

We provide some explanations for the appropriateness of the agent-based approach, choosing a narrative grounded on an historical perspective in order to contextualize the emergence of this innovative instrument. A first explanation concerns the need of having a set of cumulatively interacting agents to explain the power law distribution that are typically observed in social sciences. A second explanation concerns the possibility of designing a proper and realistic micro-foundation of macroeconomics, relaxing the unrealistic rational expectations assumption, and relying on behavioral and forecasting rules.

Finally, we present the special issue contributions, where this modelling design method has been applied to explore some of crucial challenges faced by the world economy.

## Footnotes

1 The meso dimension should be interpreted here in an evolutionary and game-theoretic frame, along the lines of Elsner and Heinrich (2009) and Elsner (2010), i.e. the study of the emergence of institutional structures at mid-sizes given certain cultural and social values that guide the economic process.

2 “is written in mathematical language, and the characters are triangles, circles and other geometric figures” (Galileo Galilei, Il Saggiatore)

3 Nelson and Winter’s simulations from the 1970s (reiterated in their 1982 book) can be considered as forerunner of the field.

4 In recent years, in the new Keynesian economics literature, models have been introduced that are able to reproduce the empirical evidence on heterogeneous responses to shocks by matching empirical distributions of income and wealth (see, e.g. Kaplan et al. (2018)). These so-called heterogeneous agent New Keynesian models (HANK) utilise heterogeneity in wealth and income to create optimal responses for various agents and aggregating them into aggregate consumption and output responses. However, it is worth noting that these new HANK models still assume equilibrium and the interactions through the price system only.

5 It is worth noting that “what-if analysis” shall not be confused here with the Friedman’s famous “as-if” argument (Friedman 1953), according to which assumptions need not be “realistic” to serve as scientific hypotheses but they merely need to make significant predictions. In particular, in this context “what-if analysis” means studying by means of computational experiments how the output of a model or system changes with respects to changes of its inputs, specifications and parameters.

6 In recent times, the fitness of ABM for describing contagion mechanisms has been exploited to model the economic impact of the COVID-19 pandemics (see, e.g. Krivorotko et al. (2021)).

7 not given/exogenous as in neoclassical “new institutional economics”, but endogenous, i.e. emerging and co-evolving, even if sometimes asynchronically with other system dimensions

8 https://www.ecb.europa.eu/press/key/date/2010/html/hsp101118.en.html

9 https://www.bankofengland.co.uk/speech/2016/the-dappled-world

10 https://www.centralbanking.com/central-banks/economics/economic-modelling/3822871/agent-based-models-a-new-frontier-for-macroeconomics

11 The famous Duhem-Quine thesis (see, e.g. Elsner et al. (2014, Cap 18)), states that it is impossible to test a single hypothesis on its own, since each one comes as part of an environment of axioms, assumptions, models, theories, auxiliary hypotheses, core examples, and empirical prototypes. Therefore, there is no verification, and no falsification even and the epistemological and theory-development process in itself is a complex system and one can find that a hypothesis, a model or some theory is highly “inappropriate”, not “fruitful”, or some measurement may “corroborate” (but never “verify”) a hypothesis. A theory is rejected in the end, by some social-scientific process through some “scientific community” (Kuhn 1962).

12 https://www.nytimes.com/2009/09/06/magazine/06Economic-t.html

13 https://voxeu.org/article/failed-forecasts-and-financial-crisis-how-resurrect-economic-modelling

14 Pierre-Simon Laplace, “Mémoire sur les approximations des formules qui sont fonctions de très-grands nombres, et sur leur application aux probabilités”, Mémoires de la Classe des sciences mathématiques et physiques de l’Institut de France, 1809, p. 353-415

15 When considering the entire size distribution, generally the distribution is log-normal (see also the Gibrat’s law (Gibrat 1931; Simon and Bonini 1958).

16 Actually, finite sample sizes do not preclude the occurrence of distributions with infinite moments under appropriate conditions. It is not possible to measure infinite moments in finite samples, but one can observe divergence of the sample moment with increasing sample size (see Yang et al. (2019)).

17 An important implications of power laws is that major events, such as crises, are not rare as foreseen by a Gaussian distribution, then black swans do occur more frequently!

18 It is also relevant to specify the kind of interactions that lead to such cumulative self-organization mechanisms, e.g. the preferential attachment (Barabasi and Albert 1999).

19 http://www.eurace.org/
